# Engineering a 3D wounded skin equivalent to study early inflammatory and regenerative responses *in vitro*

**DOI:** 10.3389/fbioe.2025.1621566

**Published:** 2025-08-22

**Authors:** Rima Nuwayhid, Nguyen Ngoc-Huyen, Dmitry Notov, Stefan Langer, Olga Kurow

**Affiliations:** ^1^ Department of Orthopaedic, Trauma and Plastic Surgery, University Hospital Leipzig, Leipzig, Germany; ^2^ Department of Upper Extremity Surgery and Microsurgery, Institute of Traumatology, Orthopedics and Plastic Surgery of Central Hospital 108, Hanoi, Vietnam

**Keywords:** 3D skin equivalent, wound healing, wound model, cytokines, *in vitro* model, bioengineered skin

## Abstract

**Introduction:**

Traditional models for studying wound healing, including 2D cell cultures and animal models, present substantial limitations in mimicking human skin physiology. In this study, we present a three-dimensional wounded skin equivalent (3DWoundSE) composed of human cells as a physiologically relevant *in vitro* platform to investigate wound healing processes.

**Methods:**

The model builds upon a previously established 3D skin equivalent (3DSE) and incorporates a reproducible partial-thickness dermal punch wound. We characterised the 3DWoundSE using histology, cytotoxicity assays, immunofluorescence staining, and pro-inflammatory cytokine profiling at multiple time points post-wounding.

**Results:**

Results revealed hallmark wound responses, including increased lactate dehydrogenase (LDH) and apoptosis-inducing factor (AIF) expression, dynamic Ki-67 proliferation changes, and a pro-inflammatory cytokine response, notably elevated IL-6, IL-8, IL-33 and TNF-α levels.

**Discussion:**

Compared to the intact 3DSE, this 3DWoundSE demonstrated enhanced responsiveness to injury and cytotoxic stimuli, confirming its utility for early wound response assessment. This platform offers a reproducible and ethically sound alternative to animal models, with potential applications in dermatological research, drug development, and therapeutic screening.

## 1 Introduction

Research in wound healing has been based on animal models and two-dimensional (2D) cell cultures. 2D cell cultures have been fundamental in biological research due to their cost-effectiveness and ease of implementation. However, this simplicity is accompanied by several notable limitations (Kapałczyńska et al., 2018). The two-dimensional growth pattern alters cellular morphology, which in turn impacts cellular differentiation and function (Meyers et al., 2006; Kilian et al., 2010). Moreover, evidence suggests that 2D culturing can significantly modify cellular biological responses and gene expression profiles (Fuchs et al., 2004; Li et al., 2006). Crucially, the two-dimensional format precludes the development of a tissue-like architecture. This means that adequate cell-cell interactions and extracellular matrix formation are not achieved, which hinders central processes such as cell signalling and protein synthesis (Pampaloni et al., 2007; Baker and Chen, 2012). The complexities of skin biology, with its intricate stratified structure, render these challenges highly relevant (Hagios et al., 1998; Chandramouly et al., 2007). Additionally, the uniform access to nutrients and oxygen experienced by cells in 2D cultures differs from the variable exposure *in vivo*, influencing their metabolism (Pampaloni et al., 2007). Collectively, these variables are likely to have a significant impact on the course and outcomes of experimental investigations.

*Ex vivo* human skin samples preserve the native skin structure, allowing the application of a variety of wounding techniques and investigative methods (Seiser et al., 2022). However, structural degradation limits the optimal experimental window to 3–4 days. Variations in donors and anatomical explant sites result in sample heterogeneity (Moniz et al., 2020). Access to human skin explants can be limited to some research institutions, as they are typically obtained during cosmetic surgery.

While *in vitro* platforms offer valuable insights, they currently cannot fully replicate the complex biological environment present in animal models. The capacity of animal models to facilitate the examination of systemic effects has contributed to their long-standing use in dermatological research, supported by well-established experimental protocols. (Dellambra et al., 2019). However, this complexity also introduces variables beyond the researcher’s control, thereby limiting reproducibility due to individual factors (Bédard et al., 2020). Furthermore, there are relevant differences in skin physiology and wound healing mechanisms between humans and animals, which likely contribute to the translational gap (Zomer and Trentin, 2018; Robinson et al., 2019). To mitigate these critical differences, human skin can be xenografted to immunocompromised mice, enabling long-term studies (Salgado et al., 2017). This method however does not address the ethical concerns around animal use and the increasing resource demands due to regulations promoting the 3R principles - Replacement, Reduction, and Refinement, whichmake the reliance on animal models increasingly difficult to justify. The 3R principles, first articulated by Russell and Burch in 1959, serve as an ethical framework for the humane use of animals in scientific research: Replacement refers to the use of alternative methods; Reduction involves strategies to minimise the number of animals used; and Refinement focuses on modifying experimental procedures to enhance animal welfare (Petetta and Ciccocioppo, 2021).

3D cell cultures offer a compelling alternative by providing a more physiologically relevant *in vitro* environment. 3D models support a native-like architecture that promotes realistic cell differentiation, signalling, and function (Duval et al., 2017). Compared to animal models, 3D cultures eliminate species-specific differences in skin physiology and wound healing, thus improving translational relevance (Langhans, 2018). Moreover, they allow for greater reproducibility by offering a controlled microenvironment without inter-individual biological variability inherent in animal studies. The ethical concerns and regulatory requirements surrounding animal testing further highlight the necessity of human-based 3D culture models in dermatological and wound healing research. By incorporating multiple cell types in an organotypical structure, 3D skin models represent a significant advancement in *in vitro* research, enabling more accurate predictions of therapeutic outcomes and disease mechanisms.

3D skin equivalents (3DSE) have been utilised to model and investigate a range of dermatological conditions and processes, including skin tumours, aging, inflammatory dermatoses, microbial infections, foreign body reactions and the effect of topical agents and their delivery (Breij et al., 2012; Kitisin et al., 2020; Liu et al., 2020; Jennen et al., 2022; Shariff et al., 2022; Ansaf et al., 2023; Aydin et al., 2024; Scheurer et al., 2024; Vázquez-Aristizabal et al., 2024).

Regardless of the model used, a variety of techniques is available to induce skin injury, including incision, excision, repeated taping, needling, drilling, burning, laser ablation, UV radiation (Egles et al., 2010; Rossi et al., 2015; Schmitt et al., 2018; Schneider et al., 2021; Seiser et al., 2022; Mulder et al., 2023a; Salazar Silva et al., 2025; Truzzi et al., 2025). To ensure uniformity, the wounding procedures can be automated (Rossi et al., 2015; Salazar Silva et al., 2025)The present study aimed to develop a 3D wounded skin equivalent (3DWoundSE), building upon a previously established 3D skin model that had demonstrated utility for evaluating the biocompatibility of implants (Nuwayhid et al., 2024). This new model was intended to provide a versatile, easily and consistently reproducible *in vitro* platform that could facilitate a range of experimental investigations into wound healing mechanisms and inflammatory responses. It provides a basis for future assessments of the cytotoxicity and efficacy of therapeutic interventions.

## 2 Materials and methods

### 2.1 Composition of 3D wounded skin equivalent

The method for fabricating a 3D skin equivalent has previously been described in detail (Nuwayhid et al., 2024). In summary, each well of a 12-well transwell insert with polymer mesh support was filled with 3.5 × 10^4^ primary human dermal fibroblasts (HDFp; pooled; CellnTec Advanced Cell Systems AG, Bern, Switzerland) embedded in 400 µL Type I rat tail collagen gel concentrated at 8.84 mg/mL (rat tail, 10 mg/mL; ibidi GmbH, Cat. No. 50201, Gräfelfing, Germany). The constructs were then incubated and equilibrated overnight in fibroblast growth medium. To enhance keratinocyte adhesion, the surface of each gel was coated with 100 µL fibronectin (Merck, F4759-1 MG; Darmstadt, Germany; 5 mg/mL in DMEM without additives). Subsequently, 3.2 × 10^5^ primary human epidermal keratinocytes (PR3D-HPEK-50; CellnTec Advanced Cell Systems AG, Bern, Switzerland) were seeded on top of each constructs in growth medium. Both HDF and HPEK were used at passage four to 8. After adhesion, culture medium was added inside and outside the inserts, and the constructs were incubated for 72 h to allow keratinocyte proliferation and surface coverage. Once a confluent keratinocyte layer formed, the constructs were elevated to an air–liquid interface and maintained in differentiation medium for 26 additional days, with media changes every 3 days. This process resulted in fully stratified, organotypic 3D skin equivalents (Nuwayhid et al., 2024).

**FIGURE 1:**
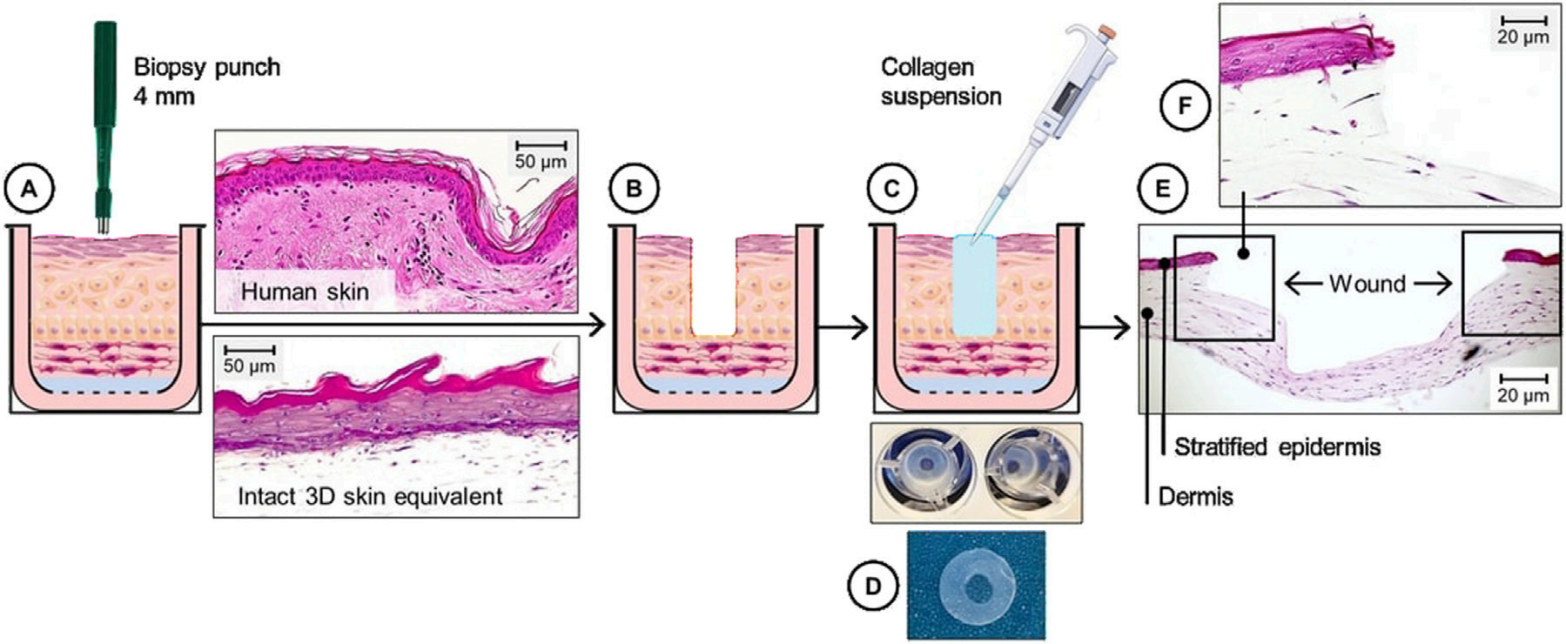
Schematic and histological overview of the composition of the 3DWoundSE. **(A–C)** A 4 mm biopsy punch was used to create a circular wound in the centre of a fully stratified 3DSE **(A,B)**, followed by **(C)** the application of a collagen type I suspension into the wound cavity to ensure structural stability. **(D)** Macroscopic aspect of 3DWoundSE in top-down view. **(E)** H&E-stained cross-section showing a stratified epidermal and dermal component surrounding the central wound area with x50 magnification. **(F)** x100 magnified view illustrates the wound margins. Scale bar = 20 μm.

To develop a 3DWoundSE, a 4 mm biopsy punch (Henry Schein Medical GmbH, Berlin, Germany) (Figure 1A) was used to manually create a central defect reaching the dermal component in 3DSE matured for 30 days (Figure 1B). To maintain the structural integrity of the model, the resulting void was then filled with a collagen gel (Figure 1C), a technique previously established (Manuela et al., 2017).

### 2.2 Histology and immunofluorescence

For histological and immunofluorescent analyses, samples of 3DSE and 3DWoundSE 24 h, 48 h and 72 h after wounding respectively were fixed in zinc-formaldehyde (Sigma Aldrich, Taufkirchen, Germany), at 4°C for 24 h, then paraffin-embedded and sectioned using a microtome onto Superfrost Plus-charged slides (Thermo Fisher Scientific, Waltham, MA, United States). The sections were subsequently deparaffinised in xylene and rehydrated with alcohol prior to standard haematoxylin and eosin staining. In the immunohistochemical experiments, the peroxidase activity was inactivated with 1% hydrogen peroxide in methanol for 10 min. Antigen retrieval was performed by boiling the slides in 0.01 M sodium citrate buffer at pH 6.0 for 10 min. Non-specific binding sites were blocked using 10% goat serum in 0.1 M phosphate-buffered saline at pH 7.4. The samples were then incubated overnight at 4°C with primary antibodies against Apoptosis-inducing factor (AIF) (Proteintech, Rosemont, IL, United States; 1:400), Kiel-67 (Ki-67) (Proteintech, Rosemont, IL, United States; 1:100), and E-Cadherin (E-Cad) (Proteintech, Rosemont, IL, United States; 1:250), diluted in the blocking buffer. After three 10-min washes in Tris-buffered saline (TBS), the slides were incubated with goat anti-rabbit Alexa Fluor 555-labelled secondary antibodies (Cell Signaling Technology, Danvers, MA, United States; 1:1,000) diluted in blocking buffer. Following another three 10-min washes in TBS, the cover slips were mounted using a medium containing DAPI (Vector Laboratories, Burlingame, CA, United States). The negative controls for the immunostaining experiments were prepared by excluding the primary antibody. Fluorescent microscopy was performed using a Leica Axiovert 100 microscope equipped with a Leica digital camera. Amount and localisation of the staining were analysed using ZEISS ZEN Blue 3.11 (Carl Zeiss Microscopy GmbH, Jena, Germany)​.

To assess the extent to which our 3DSErecapitulates native human skin in its tissue architecture, we conducted histological analyses of *ex vivo* human skin. Human skin samples were obtained from the dorsal hand of a 79-year-old female patient undergoing surgery at the Department of Plastic Surgery, University Hospital Leipzig, Germany, with ethical approval granted by the Ethics Committee of the University of Leipzig (approval number 434/20-ek). A detailed comparison between the 3DSE and *ex vivo* human skin focusing on tissue morphology, including stratification and cell-cell-contacts, has been published previously (Nuwayhid et al., 2024).

### 2.3 Cytotoxicity

LDH levels serve as an indirect marker of cell membrane integrity loss and were assessed to quantify cytotoxicity as a percentage of cell death. Culture supernatants beneath the inserts of the 3DSE and the 3DWoundSE were collected at 24 h, 48 h, and 72 h following the wounding of the 30-day-matured co-cultures and LDH activity measured using a Cytotoxicity Detection Kit (Merck KGaA, Darmstadt, Germany). Samples of both 3DSE and 3DWoundSE were treated with PBS (0% toxicity) to serve as negative controls, or with 5% Triton™ X-100 (100% toxicity) (Sigma Aldrich, Taufkirchen, Germany) as positive controls. The 100% cytotoxicity control was established by incubating the cultures with 5% Triton™ X-100 for 30 min, resulting in complete membrane lysis.

### 2.4 Cytokine response

To assess the inflammatory response following wounding, we quantified key pro-inflammatory cytokines interleukin-1 alpha (IL-1α), interleukin-6 (IL-6), interleukin-8 (IL-8), interleukin-33 (IL-33), and tumour necrosis factor-alpha (TNF-α) in the culture supernatants of 3DSE and 3DWoundSE at 24 h, 48 h, and 72 h post-wounding. using the following DuoSet ELISA kits from R&D Systems (Minneapolis, MN, United States): Human IL-1α/IL-1F1 (DY200-05), Human IL-6 (DY206-05), Human TNF-α (DY210-05), Human IL-8/CXCL8 (DY208-05), and Human IL-33 (DY3625B-05), according to the manufacturer’s instructions.

### 2.5 Statistical analysis

ELISA and cytotoxicity assay data were statistically analysed using GraphPad Prism 8.4.3 (GraphPad Software, United States). The analysis of AIF and Ki-67+ cells, as well as the intensity of E-Cad in the region of interest (ROI), was performed with ZEN Blue software, version 3.1 (Carl Zeiss Microscopy GmbH, Jena, Germany). All experiments were independently repeated at least three times, with a minimum of three samples (each originated from a different well) per group in each analysis. This resulted in a total of at least nine samples per group for cytokine and LDH measurements. Due to occasional suboptimal histological sections or stainings, a minimum of seven valid samples per group was ensured for each histological/immunofluorescence analysis. Results are presented as means ± standard deviations. Statistical significance was determined using t-tests to compare mean values, with p < 0.05 considered statistically significant.

## 3 Results

### 3.1 Histology

**FIGURE 2:**
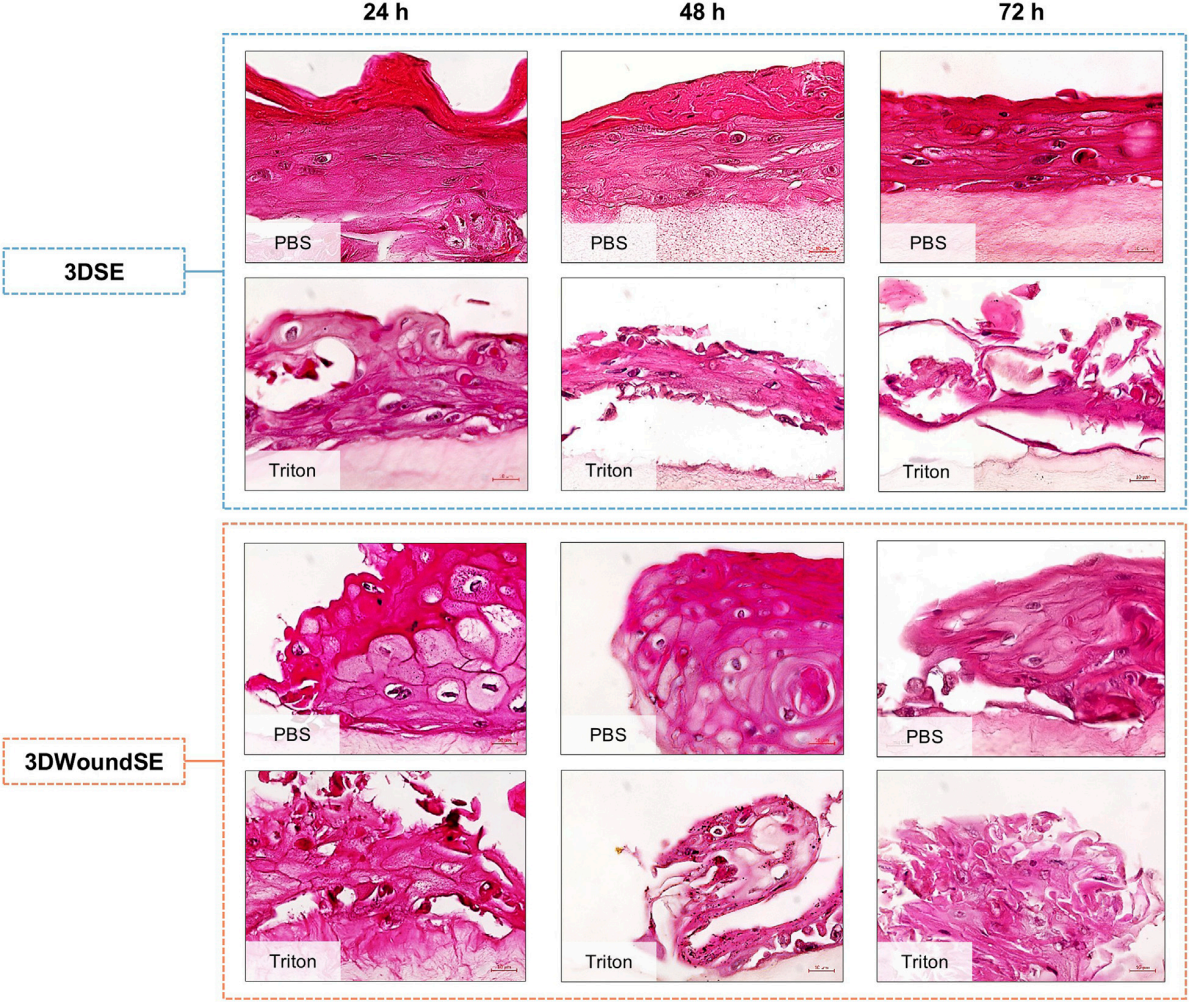
H&E staining of 3DSE and 3DWoundSE. Treatment with PBS (0% cytotoxicity, negative control) or Triton X-100™ (100% cytotoxicity, positive control). Representative histological sections show well-stratified epidermal layers in untreated 3DSE and 3DWoundSE controls (PBS) up to 72 h post-wounding, while Triton-treated samples exhibit varying degrees of epidermal and dermal disruption. All histological examinations were independently performed in triplicate, each using three biological replicates (n = 3) per group. After exclusion of samples of suboptimal quality; a minimum of seven (n = 7) valid samples per group was included in each analysis. Scale bars: 20 µm.

In histological analysis of PBS-treated 3DSE samples a stratified epidermis was evident, with a dense dermal compartment resembling native skin architecture (Figure 2). In contrast, Triton X-100 treatment disrupted epidermal integrity in the 3DSE, resulting in thinning of the epidermal layer, cellular detachment, and disorganisation of organotypic architecture across all replicates. The 3DWoundSE samples exhibited the creation of a wound with complete removal of the epidermal layers, reaching the dermal component (Figures 1E,F; Figure 2). The wound margins were composed of a fully stratified dermal layer. Triton treatment of 3DWoundSE induced a more severe tissue damage than in 3DSE, with loss of stratification, and widespread cellular disintegration.

### 3.2 Cytotoxicity

**FIGURE 3:**
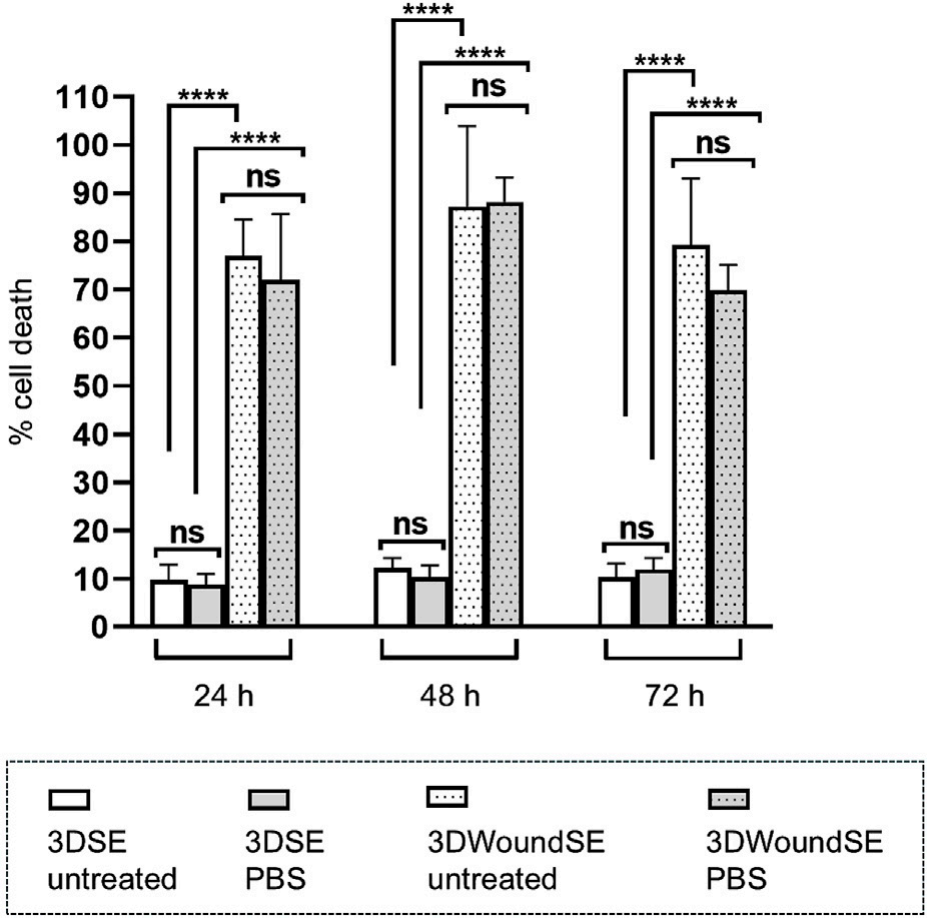
Cell death represented by LDH release from 3DSE and 3DWoundSE. Negative controls: PBS treatment or no treatment. Wounding resulted in a marked increase in LDH, indicating significant cell damage independent of treatment. In contrast, 3DSE showed minimal LDH activity across all time points. All measurements were independently performed in triplicate, each using three biological replicates (n = 3) per group, resulting in a total of nine replicates (n = 9) per group across experiments. Data are presented as mean ± SD (n = 7). ns: not significant, ****p < 0.0001.

In the 3DSE PBS-treated and untreated samples exhibited low LDH activity across all timepoints. In contrast, PBS as well as no treatment in the 3DWoundSE led to a pronounced and sustained increase in cell death represented by LDH release (p < 0.0001) (Figure 3).

### 3.3 Immunofluorescence

**FIGURE 4:**
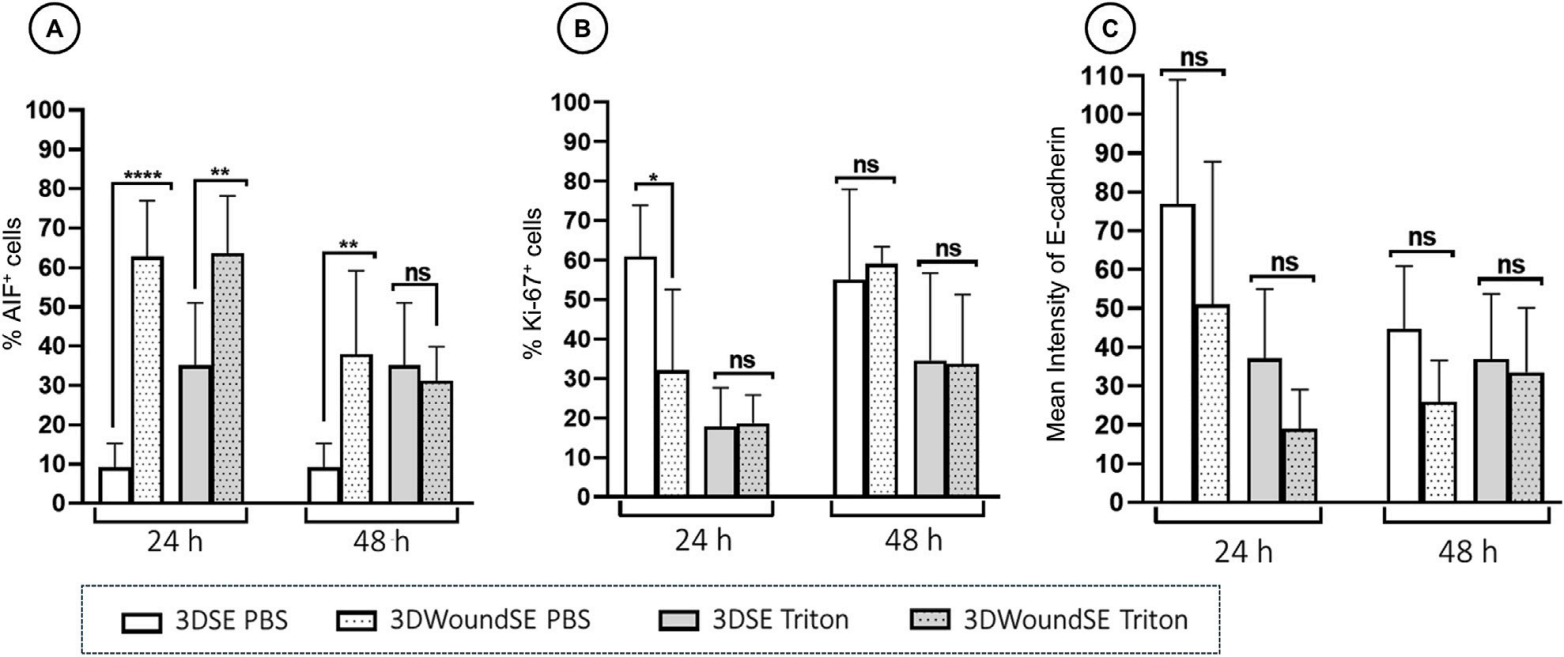
Quantitative analysis of immunofluorescence. Staining for AIF, **(A)** Ki-67 **(B)**, and E-Cadherin **(C)** in 3DSE 3DWoundSE at 24 and 48 h post-wounding. Bar graphs represent the percentage of cells stained positively for AIF and Ki-67, respectively and the mean fluorescence intensity for E-Cad, comparing Triton X-100™-treated (positive control), PBS-treated (negative control), 3DSE, and 3DWoundSE constructs. Data are expressed as mean ± SD from atleast seven (n = 7) experiments per group. ns: not significant, *p < 0.05, **p < 0.01, ****p < 0.0001.

**FIGURE 5:**
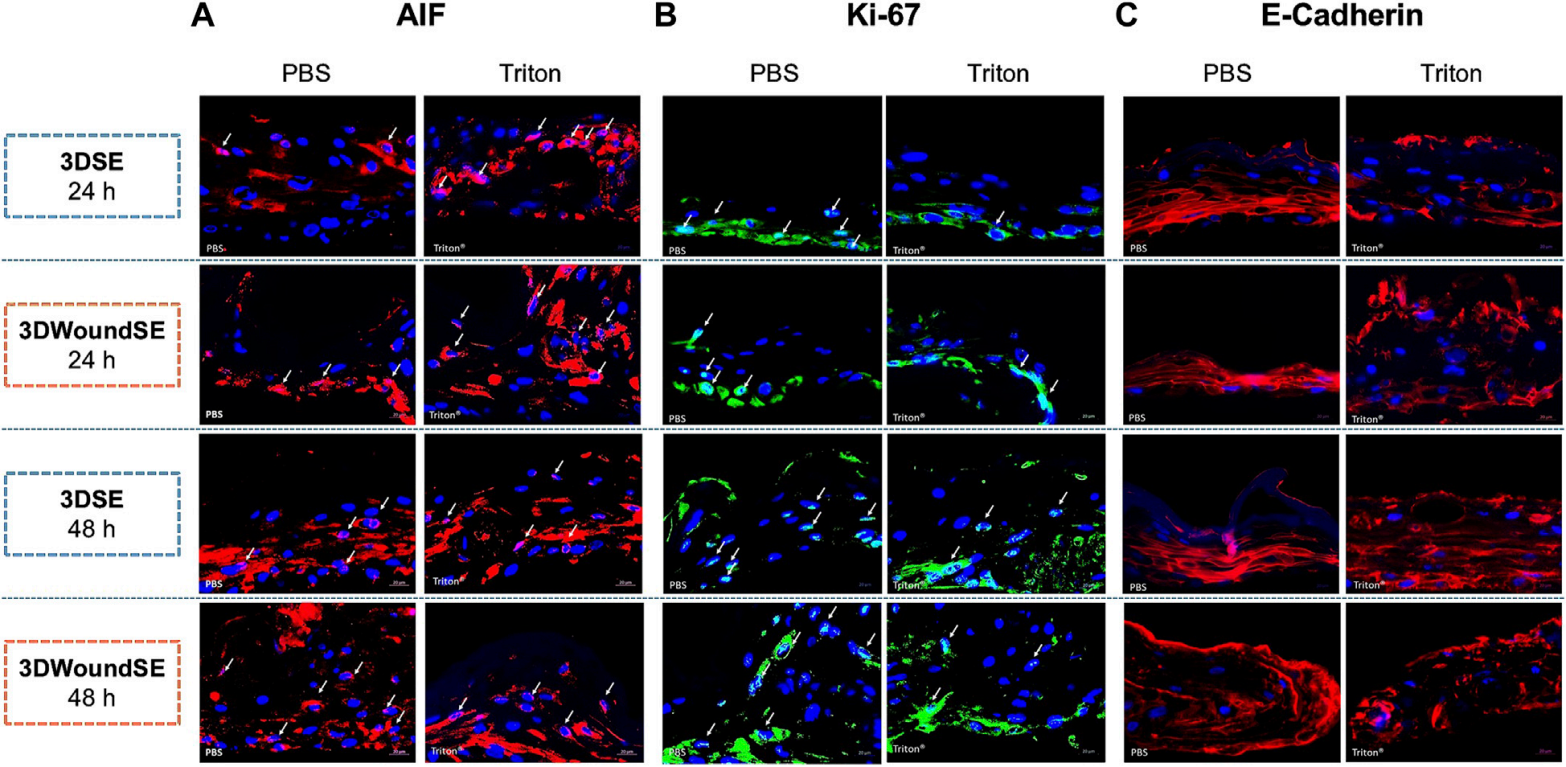
Immunofluorescence staining. Representative images show expression of apoptosis-inducing factor (AIF, pink), **(A)** proliferation marker Ki-67 (light blue), **(B)** and cell–cell adhesion protein E-Cadherin (red) **(C)** in sections of 3DSE and 3DWoundSE at 24- and 48-h post-wounding. Nuclei were counterstained with DAPI (blue). Controls treated with PBS and Triton X-100™. White arrows indicate positively stained cells. All histological examinations were independently performed in triplicate, each using three biological replicates (n = 3) per group. After exclusion of samples of suboptimal quality; a minimum of seven (n = 7) valid samples per group was included in each analysis. ×20 magnification.

**FIGURE 6:**
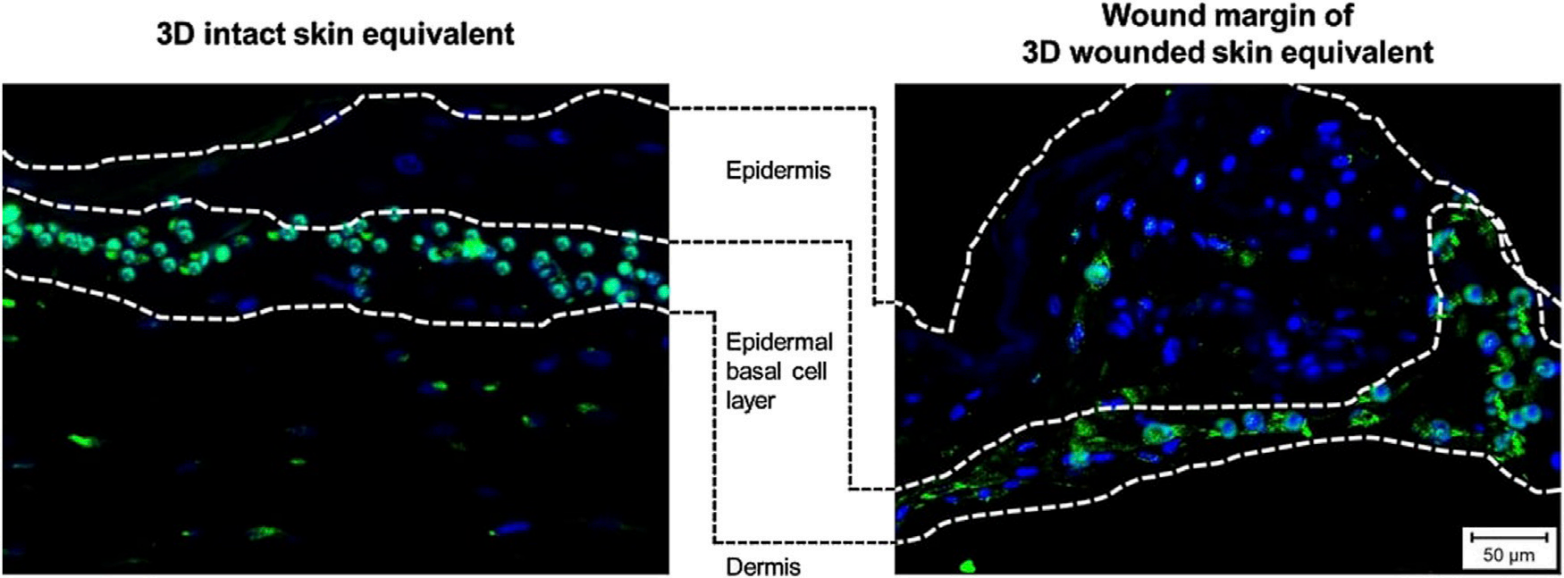
Immunofluorescence staining. Images show expression of proliferation marker Ki-67 (light blue) in sections of 3DSE (left) and the wound margins of 3DWoundSE (right), demonstrating the presence of proliferative cells in the basal and suprabasal layers of the epidermal component, indicative of re-epithelialisation.

To assess the cellular response to wounding, we performed immunofluorescence staining for apoptosis-inducing factor (AIF), Ki-67, and E-Cadherin in 3DSE and 3DWoundSE constructs at 24 and 48 h post-injury (Figures 4, 5). Consistent with visual inspection (Figure 4), the measured AIF expression was significantly elevated in 3DWoundSE at 24 h post-wounding compared to 3DSE (p < 0.0001). By 48 h, AIF levels had declined but remained higher in PBS-treated wounded constructs than in controls (p < 0.01). As anticipated, Triton treatment led to a peak in AIF-expression, with even higher levels in the 3DWoundSE (Figure 5A). Ki-67 expression, indicative of proliferative activity, was reduced in the 3DWoundSE 24 h post-injury (p < 0.05) but rose to the levels of 3DSE within the next 24 h with most activity in the basal and suprabasal layers. An increase in proliferative cells was observed in the basal and suprabasal layers adjacent to the wound edge was observed, indicating re-epithelialisation (Figure 6) Triton treated samples showed reduced Ki-67 expression, with no difference between 3DSE and 3DWoundSE (Figure 5B). E-Cadherin levels were slightly reduced in 3DWoundSE at both time points, although not statistically significant. Triton-treatment resulted in a clear reduction in cell-cell-contacts (Figure 5C).

### 3.4 Cytokine response

**FIGURE 7:**
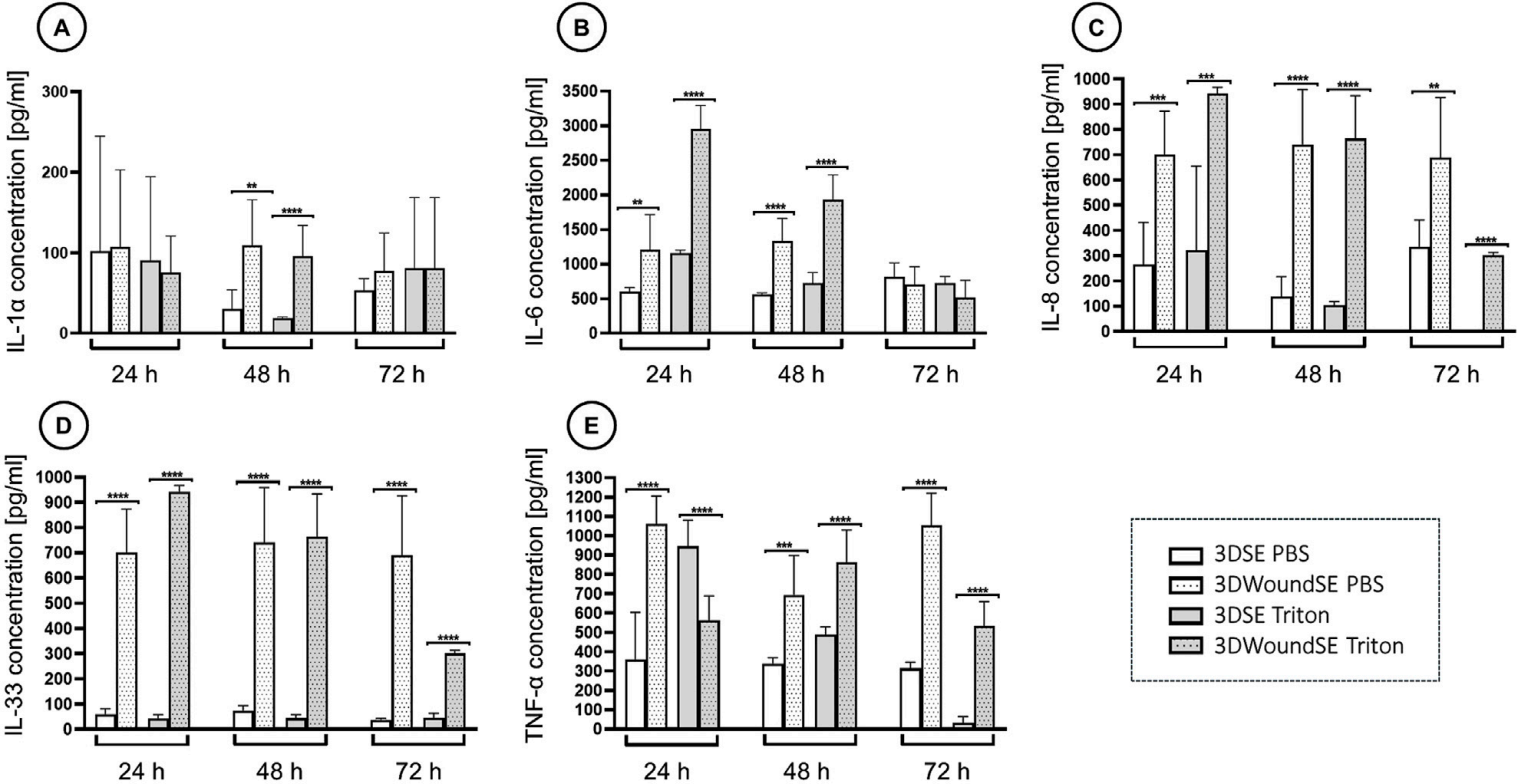
Quantification of pro-inflammatory cytokines. Concentrations of interleukin-1 alpha (IL-1α), **(A)** interleukin-6 (IL-6), **(B)** interleukin-8 (IL-8), **(C)** interleukin-33 (IL-33), **(D)** and tumour necrosis factor-alpha (TNF-α) **(E)** measured via ELISA in culture supernatants of 3DSE and 3DWoundSE at 24 h, 48 h, and 72 h post-wounding. Bars represent mean cytokine concentration ±standard deviation. Triton X-100™ served as the positive control (100% cytotoxicity) and PBS as the negative control (0% cytotoxicity). All measurements were independently performed in triplicate, each using three biological replicates (n = 3) per group, resulting in a total of nine replicates (n = 9) per group across experiments. **p < 0.01, ***p < 0.001, ****p < 0.0001.

The 3DWoundSE model exhibited strongly elevated levels of IL-6, IL-8, IL-33 and TNF-α at 24 and 48 h compared to the 3DSE, irrespective of treatment with PBS or Triton (Figure 7). While IL-1α levels at 24 h showed no significant difference between intact and wounded 3DSE, the 3DWoundSE presented with significantly higher levels by 48 h (p < 0.01) (Figure 7A). Notably, the difference was most pronounced for IL-33, with levels almost 20-fold higher (p < 0.0001) (Figure 7D). Furthermore, the Triton-treated 3DWoundSE consistently exhibited pro-inflammatory cytokine levels that were equal to or higher than those observed in the Triton-treated intact 3DSE, with the only exception being TNF-α at 24 h.

## 4 Discussion

Traditional approaches to studying skin wounds, such as 2D cell cultures and animal models, have inherent limitations. Monolayer cultures fail to replicate the complex multicellular interactions, extracellular matrix (ECM) organisation, and mechanical properties of *in vivo* skin (Sun et al., 2006; Egles et al., 2010; Duval et al., 2017; Kapałczyńska et al., 2018). Animal models exhibit species-specific differences in skin physiology, wound healing mechanisms, and immune responses, which limit their translational relevance to human conditions (Pound et al., 2004; Regenberg et al., 2009; van der Worp et al., 2010). Three-dimensional (3D) culture systems have been demonstrated to be more suitable for conducting cytotoxicity assessments, and tumour cells exhibit increased resistance to chemotherapeutic agents within 3D environments (Sun et al., 2006; Fontoura et al., 2020). These findings underscore the critical influence of tissue architecture on cellular functions. The development of a human-derived 3D model of wounded skin thus provides a physiologically more relevant platform for studying cellular dynamics, signalling pathways, and therapeutic interventions under controlled conditions.

Ensuring uniformity in wound models is essential to minimising experimental variability (Rossi et al., 2015). To establish a readily reproducible 3D model of wounded skin, we employed controlled wounding to our established 3DSE (Nuwayhid et al., 2024). In contrast to the commonly utilised techniques of scratching, tape stripping, or scalpel excision, this approach ensures the creation of a wound that is sufficiently deep to accurately mimic the complete loss of the epidermal layer while consistently generating wounds of uniform size (Egles et al., 2010; Stamm et al., 2016; Costello et al., 2023).

Histological analysis of the 3DWoundSE revealed a full-thickness skin equivalent with a partial-thickness dermal wound surrounded by completely stratified wound margins. Treatment with the detergent Triton demonstrated an overall heightened sensitivity of the 3DWoundSE to structural disruption compared to the 3DSE, supporting the potential utility for studying barrier-compromised skin. This observation was corroborated by the results of the LDH assay. While the cells in the intact 3DSE exhibited consistently low LDH release over the 72-h investigation period, the 3DWoundSE displayed much higher LDH activity. Initially, the research team was perplexed by this finding and repeated the assay with an additional negative control of completely untreated 3DSE and 3DWoundSE, yielding results nearly identical to the PBS-treated samples. We hypothesise, that the wounding process leaves injured cells at the margins of the relatively large 4-mm wound void, which constitutes a significant proportion of the overall co-culture, and the shear forces of using a biopsy punch with a twisting motion may have disrupted the integrity of cells not directly adjacent to the wound edges. This hypothesis might be supported by the slightly lower E-Cad levels observed in the 3DWoundSE, although this difference was not statistically significant, indicating transient disruption of epithelial cell–cell adhesion during the early phases of wound healing.

Furthermore, AIF activity, which represents a distinctly different pathway to cell death, exhibited a similar pattern to LDH. While PBS-treated intact 3DSE displayed low AIF levels, the PBS 3DWoundSE showed significantly elevated AIF expression, suggesting increased apoptotic activity in response to injury. The combined findings from histological examinations, LDH analyses, and AIF investigations highlight the compromised barrier function and heightened vulnerability of the 3DWoundSE, supporting its relevance for studying impaired skin conditions. Additionally, the observation that tumour cells exhibit increased resistance to chemotherapeutic agents in 3D compared to 2D culture indicates that the enhanced capacity for complex cell-cell interactions provided by 3D culture can bolster barrier functions, similar to the 3DSE’s greater resistance to the toxic agent Triton, as evidenced by lower LDH and AIF activity, as well as a less pronounced pro-inflammatory response (Fontoura et al., 2020). It must be acknowledged however, that the observed increase in LDH levels following wounding also reflects membrane disruption in the remaining cell population, rather than serving as a direct comparison to unwounded controls. Consequently, the sensitivity and relevance of LDH assays in this model are limited to assessing post-wounding cellular stress, rather than providing a measure of absolute cytotoxicity.

The damaging effects of Triton treatment and wounding both resulted in a decrease in proliferating cells as observed in the Ki-67 analysis at 24 h post-wounding. However, the increase in actively proliferating cells at 48 h indicates the model’s ability to facilitate wound healing. Specifically, the 3DWoundSE exhibited a response to wounding consistent with enhanced cellular proliferation during the early stages of the wound healing process. This also aligns with the physiological timeline of wound healing *in vivo*, where proliferation commences within 2–4 days (Landén et al., 2016; Ellis et al., 2018). This might also be reflected in the initially lowered, then again increased cell-cell-contacts marked by E-Cadherin expression. In a comparably structured model, reactions consistent with early wound healing were also observed after 48 h (Villata et al., 2024).

The non-wounded 3DSE exhibited relatively low cytokine levels throughout, consistent with the absence of a pro-inflammatory trigger and an intact barrier function. In contrast, the full panel of analysed pro-inflammatory cytokines (IL-1α, IL-6, IL-8, IL-33 and TNF-α) was induced by both destructive stimuli, wounding and Triton-treatment. IL-1α is a key initiator of the inflammatory response, stimulating keratinocyte proliferation and angiogenesis (Macleod et al., 2021). In addition to promoting leukocyte chemotaxis, IL-6 plays an important role in the transition from inflammatory to reparative response (Johnson et al., 2020). In epithelial cells, IL-6 can upregulate IL-8 levels, which in turn induces the release of chemoattractants for neutrophiles, (Schafer and Brugge, 2007; Raziyeva et al., 2021). IL-33 functions as an alarmin, released by damaged epithelial cells. It is therefore highly appropriate that the 3DWoundSE model exhibited markedly elevated levels of IL-33, given the presence of injured epithelial cells within this wounded skin equivalent. (Di Carmine et al., 2022). TNF-α, produced by fibroblasts, is an important modulator of wound healing by inducing the production of other pro-inflammatory cytokines like IL-6 and IL-8, consistent with the elevated levels in the 3DWoundSE (Osawa et al., 2002; Mahmoud et al., 2024). To our knowledge, this panel of cytokines has not been profiled in a mechanically induced, sterile 3D wound model, particularly the inclusion of IL-33 as an epithelial alarmin. However, measurements in an infected 3DWoundSE demonstrated elevated levels of IL-1α, IL-6, IL-8, and TNF-α at a 48-h time point (Villata et al., 2024), corroborating our observations. Recent work by Mulder et al. used scaffold-based full-thickness skin equivalents to evaluate cytokine release after thermal injury, demonstrating generally heightened cytokines after burn (Mulder et al., 2023a). In a subsequent study incorporating immune cells into their 3D skin model, an expanded cytokine panel revealed an adaptive immune response (Mulder et al., 2023b). While IL-6 and IL-8 were elevated in both models, our detection of a significant increase in IL-33 indicates a distinct epithelial stress response to mechanical disruption. Collectively, the cytokine response pattern validates the induction of an inflammatory environment due to wounding and the subsequent initiation of the wound healing process. This underscores the capacity of the 3DWoundSE to recapitulate key aspects of the early inflammatory response and thus providing a platform for the basic analysis of cytokine-mediated tissue repair.

In summary, LDH assay, immunofluorescence investigations and cytokine expression profiles confirm that the 3DWoundSE model captures key biological features of wound repair, including apoptosis, proliferation, and epithelial remodelling as well as its biological responsiveness. This validates its suitability for investigating early wound responses and potential modulatory effects of topical therapeutics.

A strength of our wound model lies in its 30-day maturation period, resulting in the formation of an organotypic, stratified tissue architecture, an attribute absent in other models (Iyer et al., 2018). While other standardised *in vitro* 3D wound models generate lesions confined to the epidermal layers, our 3DWoundSE introduces a deeper wound that extends to the dermal compartment (Salazar Silva et al., 2025). Villata et al. described a comparable 3DWoundSE featuring a viable, stratified epidermis after maturation for 31 days, which they utilised to explore the effects of bacterial infection (Villata et al., 2024). In contrast, our study was aimed at providing a controlled and versatile platform for evaluating inflammatory responses and the biocompatibility of topically applied therapeutic agents, thus isolating the wound response itself.

The protocol is compatible with a wide range of analytical techniques, enabling a multi-dimensional assessment of the cellular and molecular events involved in wound healing. This allows for broad applications in biomedical and pharmaceutical research. One major application is in drug discovery and development, where it allows for the testing of drugs and biomaterials in a human-relevant environment. Additionally, the model provides an ethical alternative for evaluating potential toxicity and efficacy of new treatments without the need for animal testing.

Despite the advantages of our 3DWoundSE, it has notable limitations. At this point, we have established a protocol using rat tail-derived collagen with consistent results; however, this means that our models are not exclusively human. We intend to transition to human collagen; however, a protocol with comparable reliability is still under development. One key challenge is the difficulty in fully replicating the vascularisation and immune components of human skin to further bridge the gap between *in vitro* and *in vivo* conditions. While other 3DSE have incorporated immune cells, a protocol for combining a 3D skin equivalent with a microfluidic construct has been developed to mimic perfusion and the delivery of circulating immune cells (van den Bogaard et al., 2014; Hölken et al., 2023; Mulder et al., 2023b; Hindle et al., 2025). Furthermore, a stratified epidermis has successfully been cultured atop a dermal layer containing immune cells and a capillary network, using cells isolated from *ex vivo* skin samples (Chaib et al., 2019). With a focus on keratinocyte-fibroblast signalling, our model does not include skin appendages such as hair follicles and sebaceous glands. As these structures are essential for skin architecture and function, the translational relevance of skin models remains limited until their successful integration, representing a relevant research gap (Hosseini et al., 2022). Recent advances include spheroid-transfer approaches and bioprinting ink containing precursor and stem cells. (Tan et al., 2022; Ma et al., 2025).

This study presents a reproducible, human-relevant *in vitro* organotypic wound model that effectively recapitulates key biological events of early skin wound healing, including barrier disruption, cell death, proliferation, and inflammation in a controlled environment. The 3DWoundSE has multiple potential applications in academic and preclinical context. It provides a platform for preclinical testing of topical therapeutics, assessing their effect on barrier restoration and inflammatory response. It is also suitable for evaluating re-epithelialisation and cytokine dynamics under the influence of wound dressings and biomaterials. A variety of investigative methods can be applied for basic analysis into wound healing pathologies such as delayed re-epithelialisation or inflammatory dysregulation.

Future adaptations may further increase the translational relevance of this platform. It could be modified to disease-specific parameters, e.g., a diabetic skin model. Bacterial inoculation would enable studies into infections and antimicrobial efficacy. Co-culturing with immune cells represents an essential next step to realistically represent inflammatory interactions and immune-mediated wound responses. Incorporating microfluidic platforms would advance the model toward addressing systemic factors such as immune cell recruitment or drug delivery.

Enabeling the use of a variety of investigative methods, versatile and ethically superior platform for studying the dynamics of wound repair and evaluating topical therapeutic agents.

Future integration of immune or vascular components may further enhance its translational relevance, particularly for chronic or systemic disease models.

## Data Availability

The datasets presented in this study can be found here: https://doi.org/10.6084/m9.figshare.28869569.
